# Alpha-Gal Syndrome after *Ixodes scapularis* Tick Bite and Statewide Surveillance, Maine, USA, 2014–2023

**DOI:** 10.3201/eid3104.241265

**Published:** 2025-04

**Authors:** Eleanor F. Saunders, Haris Sohail, Devin J. Myles, Dawn Charnetzky, Bryan N. Ayres, William L. Nicholson, Scott P. Commins, Johanna S. Salzer

**Affiliations:** Centers for Disease Control and Prevention, Atlanta, Georgia, USA (E.F. Saunders, B.N. Ayres, W.L. Nicholson, J.S. Salzer); University of North Carolina, Chapel Hill, North Carolina, USA (E.F. Saunders, S.P. Commins); Maine Center for Disease Control and Prevention, Augusta, Maine, USA (H. Sohail, D.J. Myles); MCD Global Health, Hallowell, Maine, USA (D.J. Myles); Maine Medical Center, Portland, Maine, USA (D. Charnetzky)

**Keywords:** vector-borne infections, zoonoses, red meat allergy, alpha-gal syndrome, Ixodes scapularis, ticks, case reports, public health surveillance, Maine, USA

## Abstract

In the United States, alpha-gal syndrome (AGS) is primarily associated with lone star tick (*Amblyomma americanum*) bites. We describe AGS onset after an *Ixodes scapularis* tick bite and present AGS surveillance in Maine, 2014–2023. US health and public health professionals should be aware of AGS outside the established lone star tick range.

Alpha-gal syndrome (AGS) is an IgE-mediated hypersensitivity to the disaccharide galactose-α-1,3-galactose (α-gal), found in tissues of nearly all mammals. Spatial distribution of AGS case-patients in the United States closely follows the geographic range of established lone star tick (*Amblyomma americanum*) populations. However, some case-patients were identified outside that range (*1*,*2*). Other global tick species cause AGS in humans; other human-biting ticks in the United States may play a role in α-gal sensitization. The α-gal molecule is found in saliva or salivary glands of *Haemaphysalis longicornis* and *Ixodes scapularis* ticks (*3*,*4*).

To examine the possible role of other tick species causing AGS in the United States, we investigated a patient with confirmed AGS in Maine, USA, showing symptoms 9 days after a blacklegged tick (*I. scapularis*) bite. The Maine Center for Disease Control and Prevention (Maine CDC) collected surveillance data for 57 confirmed or suspect cases of AGS, including this case.

## The Study

**Figure 1:**
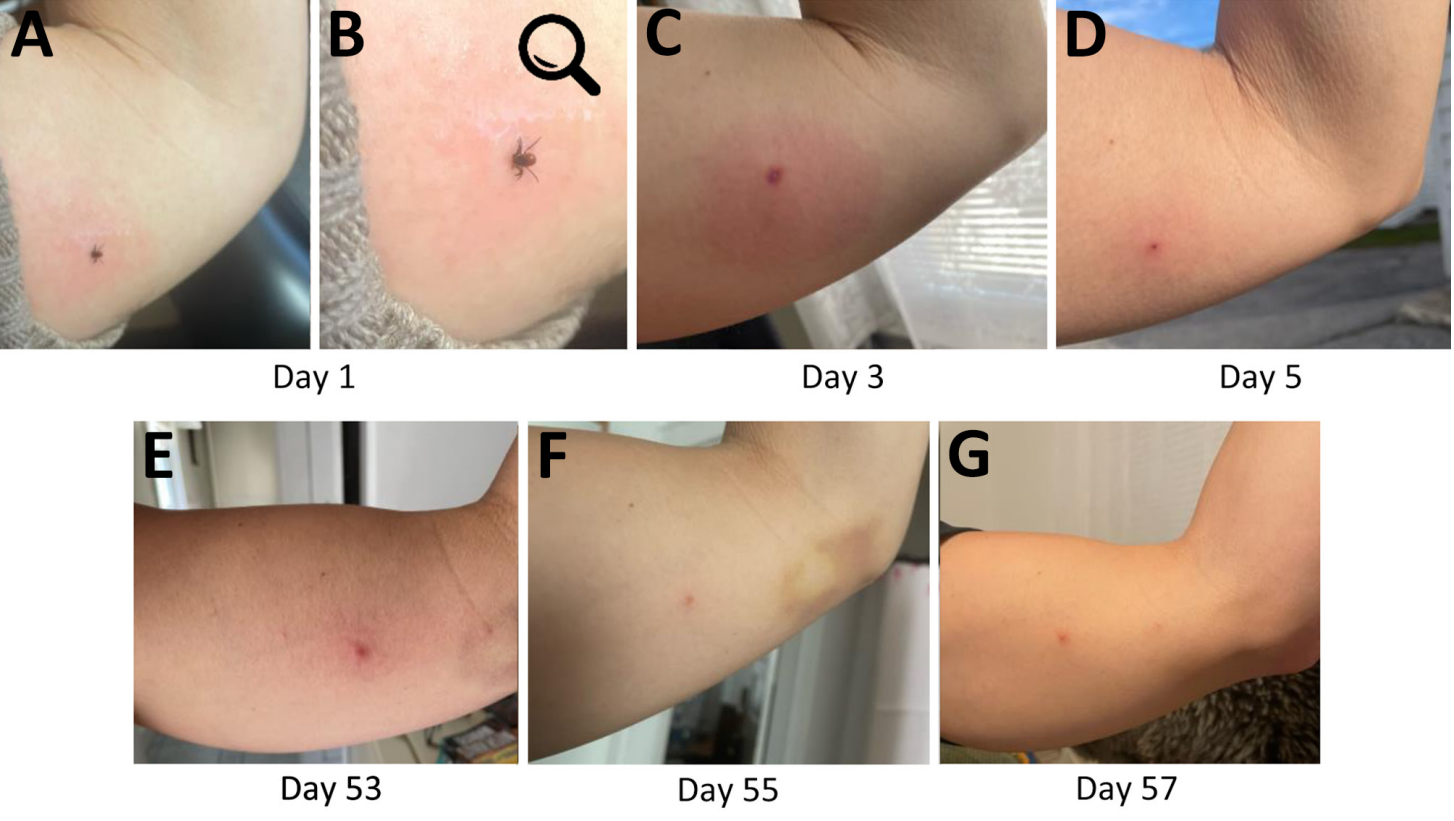
Progression and persistence of bite site reaction in alpha-gal syndrome after *Ixodes scapularis* tick bite, Maine, USA, 2022. A) Day 1, attached *Ixodes scapularis* adult female; B) enlarged version of image in panel A; original magnification ×2.1; C) day 3, growing erythema surrounding tick bite site accompanied by intense and increasing pruritus; D) day 5, bite site less inflamed and with scab; E) day 53, patient resumed photo documentation because of persistent pruritus and “flares” of lesion becoming intermittently raised; F) day 55, lesion improved; G) day 57, lesion slightly raised again, photo taken to document variation. After these photos, pruritus largely resolved. As of December 2024, the bite site remains identifiable as an asymptomatic papule.

On May 4, 2022, a 45-year-old woman discovered an attached tick medial to her left bicep after returning from a wooded path in York County, Maine (Figure 1, panel A). She appropriately removed the tick and identified it as an adult female *I. scapularis*, which entomologists at the Centers for Disease Control and Prevention (US CDC) confirmed through morphologic and molecular identification (*5*). By May 7, 2022, the bite area had become inflamed and pruritic and had an enlarged, erythematous circumference.

The patient was otherwise healthy and had no allergies and no known recent exposure to endoparasites or ectoparasites (e.g., mites) except the reported tick; she was treated 9 years earlier for presumed ascariasis (records not available) attributed to organic farm work in Maine. At the time of the tick bite, she worked indoors and participated in regular outdoor recreation in Maine. She traveled to suburban Fort Lauderdale, Florida, USA, 112 days before the tick bite and reported no known tick bites while traveling. She had not previously found an attached tick in >10 years.

The patient’s first gastrointestinal (GI) symptoms occurred May 13, 2022, nine days after the tick bite and 2.5 hours after a meal of roasted rabbit and 1 alcoholic beverage. Symptoms, including delayed-onset abdominal pain and malaise lasting ≈3 hours, continued to occur over the next 2 weeks after meals containing red meat (Table 1). All meals were shared with others who did not experience symptoms, and only mammalian meat products were associated with symptoms. A severe episode of diarrhea and vomiting hours after beef consumption prompted the patient to visit a healthcare provider (HCP) 20 days after the tick bite.

**Table 1:** Retrospective details from a patient with alpha-gal syndrome after *Ixodes scapularis* tick bite, Maine, USA, 2022*

Date	Days after tick bite	Meal	Alcohol, servings	Symptoms or clinical testing	Timing of symptoms relative to meal, h	Persons sharing meal ill
May 13	9	Roasted rabbit and vegetables	1	Abdominal pain, malaise, substernal ache, difficult concentration	2–3	No
May 14	10	Portions of ham sub, salami sub, and veal lasagna	2	Abdominal pain, malaise, substernal ache, difficult concentration	2–3	No
May 15–18	11–14	1/2 roast beef sub	0	Abdominal pain, malaise, substernal ache, difficult concentration	2–3	No
May 15	11	Chicken	0	None	NA	No
May 18	14	Fish	0	None	NA	No
May 20	16	Beef, broccoli, rice	1	Abdominal pain, diarrhea, vomiting, pain similar to prior food poisoning	2–3	No
May 21	17	Vegetarian Indian food	0	None	NA	No
May 23–27	19–23	Milkshake	0	Abdominal discomfort	2–3	NA
May 24	20			Primary care appointment, α-gal testing sent		
May 25	21	Deviled egg appetizers	2	None	NA	No
May 28	24	Pork dumplings	1	Abdominal pain, malaise, substernal ache, difficult concentration	2–3	No
June 1	28			Positive result from May 24 α-gal test		

*NA, not applicable (i.e., no symptoms).

**Figure 2:**
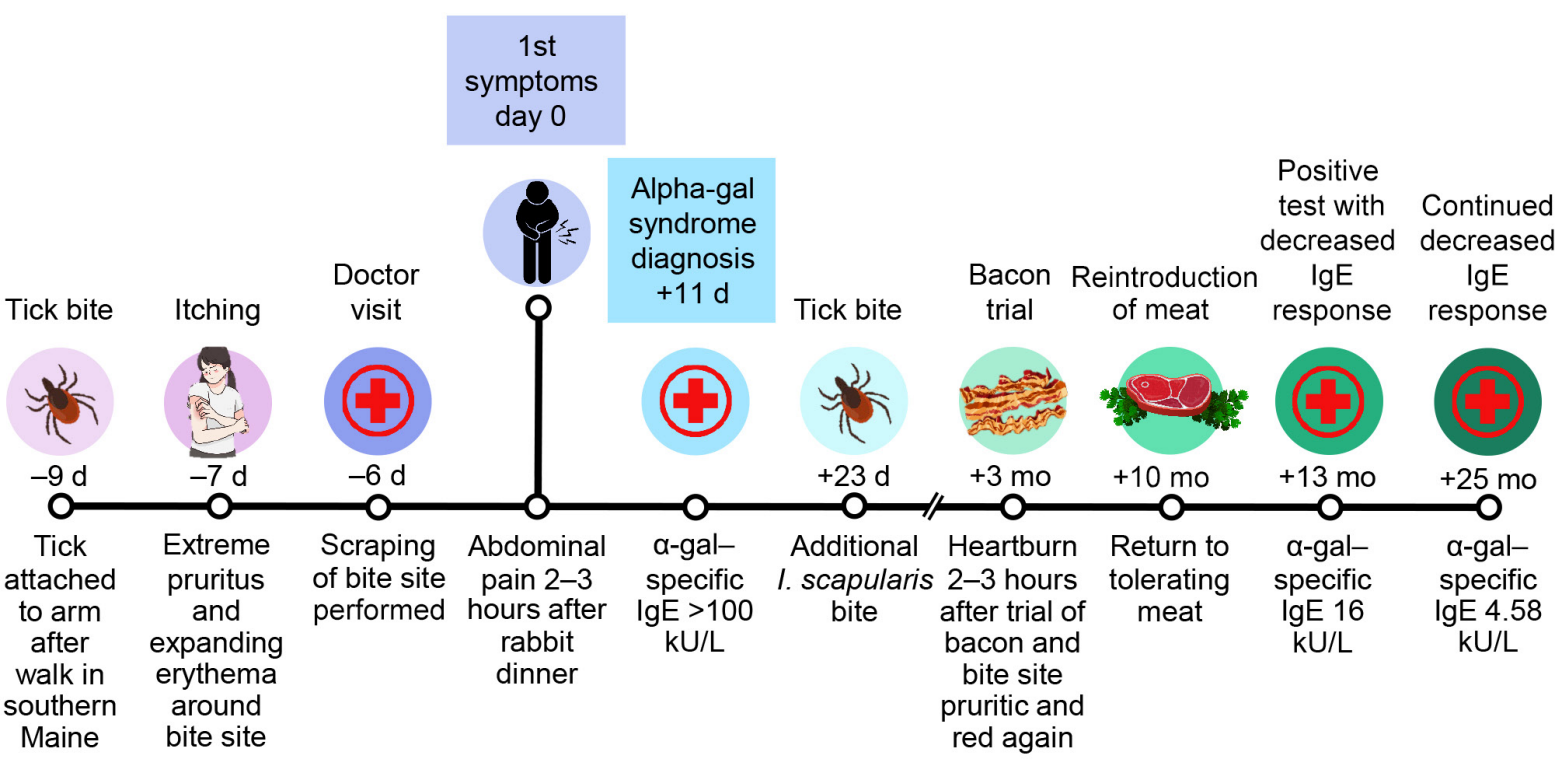
Timeline of symptom onset in a patient with alpha-gal syndrome after *Ixodes scapularis* tick bite, Maine, USA, 2022.

The HCP ordered a complete blood count, comprehensive metabolic panel, lipase, amylase, and *Helicobacter pylori* breath test on May 24, 2022, to investigate the patient’s abdominal pain. All results were unremarkable. An ultrasound for gallstones was ordered but not completed because results of serum α-gal–specific IgE testing showed levels greater than the upper limit of detection at >100 kU/L (Eurofins Viracor, https://www.eurofins-viracor.com) (Figure 2). The HCP advised avoiding beef, pork, and lamb. The patient continued to consume and tolerate small amounts of dairy, including cheese and half and half. Ice cream and milkshakes caused delayed mild GI distress and nausea. Symptoms resolved on an avoidance diet.

The patient’s symptoms fit the GI phenotype of AGS (*6*). She did not experience hives, angioedema, respiratory difficulty, hypotension, or anaphylaxis. Meal cofactors, such as alcohol and exercise, might have accentuated symptoms (*7*).

The patient found 2 ticks attached in the 1–2 months after initial tick attachment: nymphal *I. scapularis* (US CDC morphologic and molecular confirmation) and *Dermacentor variabilis* (patient identification, specimen not available). The nymphal *I. scapularis* bite site became mildly pruritic, but the *D. variabilis* bite site did not. The original tick bite site remained pruritic for 2 months.

Consumption of bacon at 3 months and steak at 7 months after first symptoms led to delayed heartburn sensations. Ten months after first symptoms, the patient tolerated a steak dinner and roast beef sandwich and resumed eating red meat. α-gal–specific IgE at 13 months (June 13, 2023) measured 16 kU/L and at 25 months measured 4.58 kU/L.

Although AGS is not a reportable condition in Maine, Maine CDC increased surveillance efforts in 2023 to elucidate epidemiologic trends in locally reported cases (Table 2). Maine CDC received positive α-gal–specific IgE laboratory reports spanning November 2014–October 2023 for 57 Maine residents (average age 57 years; range 7–81 years), including 35 (61%) men and 22 (39%) women; 29 (51%) were 45–64 years of age, and 20 (36%) were >65 years of age. Of the 57 case-patients, 55 had race and ethnicity data available and reported White race and non-Hispanic ethnicity. Case-patients resided in 12 counties; 74% lived in coastal counties (Table 2).

**Table 2:** Retrospective surveillance summary of residents with confirmed or suspect alpha-gal syndrome, Maine, USA, 2014–2023*

Category	Total no. cases/no. in category (%)
Sex	
F	22/57 (39)
M	35/57 (61)
Race	
White	55/57 (96)
Unknown	2/57 (4)
Ethnicity	
Non-Hispanic	55/57 (96)
Unknown	2/57 (4)
Suspect cases, no or insufficient clinical documentation for confirmation	34/57 (60)
Clinical diagnosis of alpha-gal syndrome, reaction timing unspecified	7/34 (21)
Clinical evidence of allergy to meat or α-gal–containing products, reaction timing unspecified	16/34 (47)
Insufficient symptom data	11/34 (32)
Confirmed cases	23/57 (40)
Interviewed by public health officials†	12/23 (52)
History of tick bite	
Recent tick bite	11/12 (92)
No known recent tick bite	1/12 (8)
Travel	
OOS travel <6 mo preceding symptom onset	3/12 (25)
No OOS travel <6 mo preceding symptom onset, OOS travel >6 mo reported	4/12 (33)
No OOS travel <6 mo preceding symptom onset, OOS travel >6 mo unknown	3/12 (25)
No travel history obtained	2/12 (17)

*Fifty-seven confirmed or suspect cases identified by Maine Center for Disease Control and Prevention, November 2014–October 2023. Mean age, 57 years; age range, 7–81 years. Cases classified as suspect or confirmed according to 2022 Council of State and Territorial Epidemiologists standardized case definition for alpha-gal syndrome. Thirty-four of 57 persons with suspected cases could not be interviewed, and medical charts lacked information about symptom timing required to meet confirmed case definition. OOS, out of state. †Another 11/23 case-patients confirmed through chart review or provider interview only.

Maine CDC confirmed AGS in 23 of 57 case-patients according to the 2022 Council of State and Territorial Epidemiologists case definition by interviewing HCPs or patients and reviewing medical charts. The other 34 case-patients were categorized as suspected cases. Those cases were not confirmed because of insufficient information in medical charts and absence of patient interviews. Insufficient information took 1 of 2 forms: not enough information to confirm allergy (11/57 case-patients) or evidence of red meat allergy noted but without delayed timing of symptoms specified (23/57 case-patients). For the 23 case-patients without delayed timing of symptoms specified, chart review revealed clinical evidence of allergy to meat or α-gal–containing products, including 7 case-patients with clinician-diagnosed AGS.

Interviews were conducted with 12 of 23 confirmed case-patients and travel histories collected from 10. Regarding symptom onset date, 3 of 10 reported and 7 of 10 denied out-of-state travel in the prior 6 months, but 4 of 7 who denied proximate travel reported out-of-state travel or residence in prior years. Of 12 case-patients interviewed, 11 reported >1 recent tick bites. Interviewed case-patients self-reported exposures to* A. americanum*, *I. scapularis*, and other ticks. Chart review and HCP interviews did not capture travel or tick exposures for the 11 confirmed case-patients not interviewed.

## Conclusions

We report a confirmed case of AGS in a Maine resident with symptom onset 9 days after a confirmed bite from an *I. scapularis* tick. Although lone star ticks have been sporadically found in Maine since the 1990s, established populations have not been identified (*8*). Lone star ticks comprised 0.5% of specimens submitted to the University of Maine Cooperative Extension Tick Laboratory in 2022 (*9*). Of 95 lone star tick submissions since 2019, at least 20% were reportedly acquired in Maine (*10*). The patient described here was bitten early in tick season, and the bite produced a pronounced cutaneous reaction often described in AGS, suggesting the *I. scapularis* tick bite on May 4, 2022, was associated with AGS onset (*7*,*11*,*12*).

A prior study found that patients with AGS report greater numbers and frequency of tick bites than do negative controls (*13*). The patient might have experienced other lifetime tick bites, some unrecognized, that contributed to AGS development. Prior α-gal sensitization from other lifetime tick bites or prior *Ascaris* roundworm exposure (associated with α-gal sensitization globally) might also explain the rapid 9-day symptom development (*14*,*15*). Nine days is within a range observed in coauthor experiences diagnosing and managing patients with AGS (S.P. Commins, unpub. data).

In conclusion, we confirmed AGS in 23 Maine residents identified through retrospective surveillance. Further exploration is necessary regarding the role of *I. scapularis* ticks in AGS and factors driving AGS onset in patients residing outside the established lone star tick range. Our findings highlight the need for HCPs and public health professionals in such regions to be aware of AGS.
